# Pure sensory stroke from compression of putaminal haemorrhage: a case report

**DOI:** 10.1186/1757-1626-2-34

**Published:** 2009-01-09

**Authors:** Daoming Tong

**Affiliations:** 1 Department of Neurology, the Affiliated Pingxiang Hospital, Southern Medical University, PingXiang 337000, PR China

## Abstract

**Introduction:**

The literature rarely describes putaminal haemorrhage producing pure spinothalamic sensory deficit. Here reports a case of putaminal haemorrhage in which selective impairment of the spinothalamic sensory modality was due to the compression of the hematoma.

**Case presentation:**

A 57 year old hypertensive man presented with a pure sensory stroke(PSS), and CT scan showed a putaminal haemorrhage. The clinical course was characterized by rapid resolution of the deficits.

**Conclusion:**

This case illustrates this rarely of PSS from compression of putaminal haemorrhage of good functional and vital prognosis, and stresses the value of CT scanning for diagnosis and prognosis.

## Introduction

Pure sensory stroke is a well-defined clinical entity in which hemisensory symptoms predominate without other major neurological signs [1]. Most patients were caused by a lacunar syndrome, either infarction or haemorrhage. However, the literature rarely describes putaminal haemorrhage producing pure spinothalamic sensory deficit [2]. Here reports another case of putaminal haemorrhage in which selective impairment of the spinothalamic sensory modality was due to the compression of the hematoma.

## Case presentation

**Figure 1:**
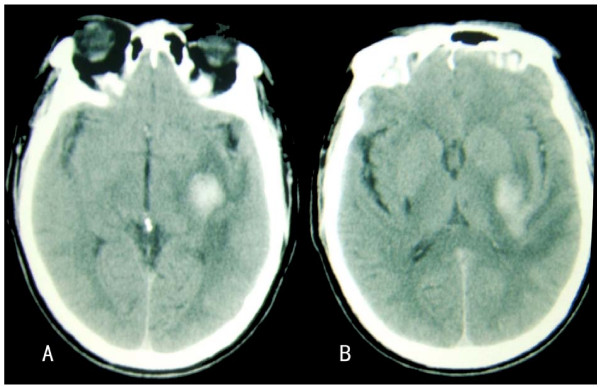
**(A) *CT scan at a level corresponding to the *mid-thalamus, showing the hemorrhage located laterally to the posterior limb of the internal capsule**. (B) *CT scan with hemorrhage at the level of the left *putamen, its posterior aspect is adjacent to the posterior part of the posterior limb of the internal capsule, and the posterior horn of the lateral ventricle compressed.

A 57-year-old man with a history of hypertension and cerebral infarction was admitted due to suddenly of numbness on his right hand, arm, leg, and face. He had no headache, vomiting, and other symptoms. On admission, his blood pressure was 180/100 mmHg. He was alert with normal cranial nerves, motor functions, and speech. There was decreased touch and pinprich sense in the right half of his body that worsened in the lower extremity. Position and vibration sense were normal in the right fingers and toes, and stereognosis and graphesthesia were normal. The deep tendon reflexes were normal, and plantar reflexes were flexor. Coordination was intact bilaterally. Routine laboratory tests were normal. Brain computed tomographic scan showed a small hematoma in the left putaminal region occurring in the territory of the posterior branches of lateral lenticulostriate arteries, the area of decreased attenuation surrounding the hematoma represents edema and the posterior horn of the lateral ventricle compressed (Fig 1.A, B). No blood was detected in cuts at the level of the posterior limb of the internal capsule. The volume of this hematoma was estimated to be 6.3 cu mm. Mannital 20% was given in a dose of 0.5 g/kg IV infusion and repeated q8h. He was discharged with his symptom and sign completely resolved after 8 days.

## Discussion

Hiraga et al [2] described putaminal haemorrhage producing pure spinothalamic sensory deficit, and Kim [3] described 3 patients who had a hypertensive putaminal haemorrhage with pure sensory stroke. The sensory symptoms were marked and persistent in those patients, which suggested that thalamocortical sensory pathways were exclusively involved. Groothius et al [4] indicated that to produce this PSS in capsular, the lesion should occupy the posterior quarter of the posterior limb of the internal capsule. In present report, my patient CT scan show a hematoma around a low density edema in the left putamina(Fig. 1. A, B). No blood was detected in cuts at the level of the posterior limb of the internal capsule. However the patient had modest superficial sensation impairment on the right side of body without deep sensation and motor function put into trouble, his symptom and sign were completely resolved after several days. We speculated, thus, the transient sensory imparment may be caused by the hematoma and its border of edema selective to compress the spinothalamic sensory tract in the posterior part of the posterior limb of the internal capsule, rather than actual destruction. The rarity of our observation shows that the size and location of the hematoma fell into the lowest portion of the spectrum of putamen, in particular, its lateral location along the posterior aspects of the putamen was responsible for pressure effects of the adjacent the posterior psrt of the internal capsule posterior limb(Fig. 1.B). This CT scan sign has been correlated with good functional prognosis, as indicative of lack of extension of the bleed across the posterior part of the internal capsule [5]. These anatomical features explain the clinical presentation as PSS.

## Conclusion

This case illustrates this rarely of PSS from compression of putaminal haemorrhage of good functional and vital prognosis, and stresses the value of CT scanning for diagnosis and prognosis.

## Abbreviations

PSS: Pure sensory stroke; CT: Computed tomographic scan; IV: Intra venous

## Consent

Written informed consent was obtained from the patient for publication of this case report and accompanying images. A copy of the written consent is available for review by the Editor-in-Chief of this journal.

## Competing interests

The author declares that he has no competing interests.
